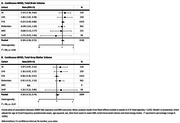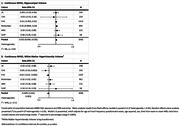# Higher MIND diet scores are associated with larger brain MRI hippocampal volume: the cross‐cohort collaboration

**DOI:** 10.1002/alz.086473

**Published:** 2025-01-09

**Authors:** Debora Melo van Lent, Daniel Koijs, Josh Bis, Robin Buelow, Tosca O.E. de Crom, Charles Decarli, Carole Dufouil, Hans J. Grabe, Leslie Grasset, Saptaparni Ghosh, Monica Goss, Stefan Frenzel, Jayandra J. Himali, Arfan Ikram, Oscar L. Lopez, Thomas H. Mosley, Ramachandran S Vasan, Cécilia Samieri, Claudia L Satizabal, Jeannette Simino, Aline Thomas, Meike W. Vernooij, Henry Voelzke, Trudy Voortman, Frank J. Wolters, Amber Yaqub, Sudha Seshadri, Alexa S Beiser

**Affiliations:** ^1^ University of Texas Health Science Center at San Antonio, San Antonio, TX USA; ^2^ Framingham Heart study, Framingham, MA USA; ^3^ Glenn Biggs Institute for Alzheimer’s & Neurodegenerative Diseases, University of Texas Health San Antonio, San Antonio, TX USA; ^4^ Boston University, Boston, MA USA; ^5^ The Framingham Heart Study, Framingham, MA USA; ^6^ Department of Biostatistics, Boston University School of Public Health, Boston, MA USA; ^7^ Boston University School of Public Health, Boston, MA USA; ^8^ University of Washington, Seattle, WA USA; ^9^ University Medicine Greifswald, Greifswald, Mecklenburg‐Voor‐Pommeren Germany; ^10^ Erasmus University Medical Center, Rotterdam, Zuid Holland Netherlands; ^11^ University of California, Davis School of Medicine, Sacramento, CA USA; ^12^ Center for Neuroscience, University of California at Davis, Sacramento, CA USA; ^13^ Centre INSERM U1219, Institut de Santé Publique, d'Epidémiologie et de Développement (ISPED), Bordeaux School of Public Health, Université de Bordeaux, Bordeaux France; ^14^ University Medicine Greifswald, Greifswald Germany; ^15^ Centre INSERM U1219, Institut de Santé Publique, d'Epidémiologie et de Développement (ISPED), Bordeaux School of Public Health, Université de Bordeaux, Bordeaux, FL France; ^16^ Boston University Chobanian & Avedisian School of Medicine, Boston, MA USA; ^17^ University of Texas Health San Antonio, San Antonio, TX USA; ^18^ Department of Population Health Sciences, University of Texas Health Sciences Center, San Antonio, TX USA; ^19^ Glenn Biggs Institute for Alzheimer’s & Neurodegenerative Diseases, University of Texas Health Science Center, San Antonio, TX USA; ^20^ University of Pittsburgh Alzheimer’s Disease Research Center, Pittsburgh, PA USA; ^21^ University of Mississippi Medical Center, Jackson, MS USA; ^22^ Boston University School of Medicine, Boston, MA USA; ^23^ The University of Texas School of Public Health San Antonio, San Antonio, TX USA; ^24^ Bordeaux Population Health Research Center, Inserm U1219, University of Bordeaux, Bordeaux France; ^25^ The University of Texas Health Science Center at San Antonio, San Antonio, TX USA; ^26^ Glenn Biggs Institute for Alzheimer’s & Neurodegenerative Diseases, University of Texas Health Science Center at San Antonio, San Antonio, TX USA; ^27^ Boston University and the NHLBI’s Framingham Heart Study, Boston, MA USA; ^28^ Taub Institute for Research on Alzheimer’s Disease and the Aging Brain, Columbia University, New York, NY USA; ^29^ Univ. Bordeaux, Inserm, BPH, U1219, Bordeaux France; ^30^ Department of Epidemiology, Erasmus University Medical Center, Rotterdam Netherlands; ^31^ Department of Radiology and Nuclear Medicine, Erasmus University Medical Center, Rotterdam Netherlands; ^32^ Department of Radiology & Nuclear Medicine and Alzheimer Center, Erasmus MC, Rotterdam Netherlands; ^33^ Harvard T.H. Chan School of Public Health, Boston, MA USA; ^34^ Glenn Biggs Institute for Alzheimer’s & Neurodegenerative Diseases, University of Texas Health Sciences Center at San Antonio, San Antonio, TX USA

## Abstract

**Background:**

Higher Mediterranean‐ DASH for Neurodegenerative Delay (MIND) diet scores have previously been associated with larger total brain volume (TBV) in the Framingham Offspring Study (FOS) community‐based cohort. We investigated cross‐sectional relationships between the MIND diet and structural brain imaging volumes and white matter hyperintensity volume (WMHV) across six community‐based cohorts.

**Method:**

We analyzed data from 3130 dementia‐, stroke‐ and other neurological disease free adults (aged 65 to 74) who participated in the Atherosclerosis Risk in Communities (ARIC) cohort, Cardiovascular Health Study (CHS), Three City (3C) cohort, FOS cohort, Rotterdam Study (RS) or the Study of Health in Pomerania (SHIP) cohort. Individuals completed a brain magnetic resonance imaging (MRI) scan, and a validated food frequency questionnaire (FFQ) (ARIC, CHS, FOS, RS), 24h dietary recall (3C), or an extensive food list (SHIP). The MIND diet consists of ten healthy (e.g. green leafy vegetables, berries and fish) and five unhealthy (e.g. cheese, red meat and products and fast fried foods) components. Outcomes from brain MRI included TBV, total grey matter volume (TGMV), hippocampal volume (HPV), and WMHV. We used multivariable linear regression to relate MIND diet adherence to the outcomes. Results were combined in meta‐analysis using fixed effects and random effects models.

**Result:**

Higher MIND diet scores (score range: 0‐15) were associated with larger HPV (beta = 0.015, 95% confidence interval = 0.004 to 0.026, cm³ per one unit MIND diet score increase) after adjustment for age, age squared, sex, time from clinical exam to brain MRI exam, total intracranial volume and energy intake, but not with TBV, TGMV and WMHV. Heterogeneity between studies was low (I^2^ = 0% TBV, TGMV, HPV) to moderate (I^2^ = 44% WMHV).

**Conclusion:**

In cross‐sectional analyses, higher MIND diet scores were associated with larger HPV, but not with other brain volume measures. It might be that HPV was a more sensitive marker of brain health in the populations under study. Future studies are encouraged to examine the associations between the MIND diet and amyloid and tau positron emission tomography (PET) imaging to elucidate whether a relationship between the MIND diet and dementia pathologies exists.